# Transverse dental arch relationship at 9 and 12 years in children with unilateral cleft lip and palate treated with infant orthopedics: a randomized clinical trial (DUTCHCLEFT)

**DOI:** 10.1007/s00784-015-1451-2

**Published:** 2015-04-10

**Authors:** R. L. M. Noverraz, M. A. Disse, E. M. Ongkosuwito, A. M. Kuijpers-Jagtman, C. Prahl

**Affiliations:** 1 Department of Orthodontics, Academic Centre for Dentistry Amsterdam (ACTA), Gustav Mahlerlaan 3004, 1081 LA Amsterdam, The Netherlands; 2 Department of Orthodontics and Craniofacal Biology and Cleft Palate Craniofacial Centre, Radboud University Medical Centre, Nijmegen, The Netherlands

**Keywords:** Cleft palate, Treatment outcome, DUTCHCLEFT, Unilateral cleft lip and palate (UCLP), Infant orthopedics, Transverse dental arch relationship

## Abstract

**Objective:**

A long-term evaluation to assess the transverse dental arch relationships at 9 and 12 years of age in unilateral cleft lip and palate treated with or without infant orthopedics (IO). The hypothesis is that IO has no effect on the transverse dental arch relationship.

**Material and methods:**

A prospective two-arm randomized controlled trial (DUTCHCLEFT) in three academic cleft palate centers (Amsterdam, Nijmegen and Rotterdam, the Netherlands). Fifty-four children with complete unilateral cleft lip and palate and no other malformations were enrolled in this evaluation. One group wore passive maxillary plates (IO+) during the first year of life, and the other group did not (IO−). Until the age of 1.5, all other interventions were the same. Hard palate was closed simultaneously with bone grafting according to protocol of all teams. Orthodontic treatment was performed when indicated. The transverse dental arch relationship was assessed on dental casts using the modified Huddart/Bodenham score to measure the maxillary arch constriction at 9 and 12 years of age.

**Results:**

No significant differences were found between the IO+ and IO− groups. Differences between the centers increased from 9 to 12 years of age.

**Conclusions:**

Transverse dental arch relationships at 9 and 12 years of age do not differ between children with UCLP treated with or without IO.

**Clinical relevance:**

There is no orthodontic need to perform IO as applied in this study in children with UCLP.

## Introduction

According to the Dutch Association for Cleft Palate and Craniofacial Anomalies (NVSCA), one infant in every 1000 is born worldwide presenting a unilateral cleft of the lip and/or palate (UCLP). In the multidisciplinary treatment of children with UCLP, infant orthopedics (IO) was introduced in the early 1950s of the last century in order to improve maxillary arch form to mainly facilitate surgery. A narrow, well-aligned cleft would be easier to repair with less undermining and mobilization of soft tissues. A narrow cleft would also lead to less tension on the repaired lip reducing scar tissue formation [1–3]. Proponents state that besides esthetical and anatomical advantages, the need for secondary surgeries is reduced [4–7], and feeding and speech development were improved [8–12]. Last but not least, IO was also thought to support the parents’ active role in the care for their child [12, 13].

Since McNeil, many derivates of the initial appliance have been introduced, but the effectiveness even up to this day remains a subject of controversy in literature. Two recent systematic reviews concluded that existing evidence cannot support the short- or long-term effectiveness of IO in UCLP patients due to the variety of treatment outcomes in RCTs, small sample sizes, and missing information on sample selection [14, 15].

From these reviews, it was concluded that DUTCHCLEFT was the only study with a proper methodological design. The results of this trial indicated that there is no evidence that IO improves feeding, general body growth, parents’ satisfaction, esthetical outcome, maxillofacial growth, speech, and language development during the first 6 years of life. A cost-effectiveness analysis that was part of the trial showed that IO was not cost-effective [16] and has no observable effect on the transverse occlusion and maxillary arch dimensions in the deciduous dentition at 4 and 6 years of age [17–19].

This study is part of the DUTCHCLEFT trial, aiming to investigate the long-term effect of IO on the transverse dental arch relationship in children with complete UCLP at 9 and 12 years of age. The hypothesis is that IO has no effect on the transverse dental arch relationship.

## Material and methods

### Subjects

A detailed description of the experimental design, treatment assignment, treatment protocol, and operators used in this study can be found in Prahl et al. [13]. A summary of the most important issues is given below.

The experimental design was a prospective two-arm randomized controlled clinical trial in three participating academic cleft palate centers in the Netherlands: Amsterdam, Nijmegen, and Rotterdam. The Institutional Review Board of each of the three centers approved the study protocol. Entrance of the trial was during the years 1993–1996. Sample size calculation was based on the detectable IO effect (3°) on the angle Sella-Nasion-Point A (SNA) at the age of 4 years. The minimum number of participants was calculated at 23 per group. The inclusion criteria were complete UCLP, infants born at term, both parents Caucasian and fluent in the Dutch language, and trial entrance within 2 weeks after birth. The exclusion criteria were soft tissue bands and other congenital malformations. When the parents agreed to participate in the study, they were asked to provide written informed consent. Between 3 and 6 months of age, all included children, 41 boys and 13 girls, were checked by the geneticist of their own cleft lip and palate team and were classified as being nonsyndromic.

Treatment assignment was concealed, and a computerized balanced allocation method [20] was used in order to reduce imbalance on relevant prognostic factors between the IO+ and IO− groups. Patients were allocated based on birth weight (<3300 or ≥3300 g) and alveolar cleft width (<8 mm, between 8 and 12 mm, or >12 mm). The allocation ratio for the IO+ and IO− groups was 1:1. The orthodontists were the only caregivers not blinded for the treatment with or without IO. One investigator (CP) controlled three individual computer programs containing the information on respectively center 1, center 2, and center 3.

### Treatment

In order to standardize treatment, all participating specialists joined consensus meetings. Until the age of 1.5 years of age, consensus was reached on timing and type of the surgical interventions and the surgeons standardized their surgical techniques. There were no changes to methods after trial commencement. Lip surgery was performed at 18 weeks of age according to the Millard technique. The soft palate was closed at the age of 52 weeks according to a modified von Langenbeck procedure. Around 9 years of age, the hard palate was closed in combination with alveolar bone grafting (Table 1). Orthodontic treatment was performed when indicated.

**Table 1:** Treatment protocols for patients with a complete unilateral cleft lip and palate from birth until 12 years of age of the cleft palate centers in this study

Timing	Center 1	Center 2	Center 3
Birth	IO Mean duration 13.4 months, SD 3.24	IO Mean duration 9.2 months, SD 4.07	IO Mean duration 13.0 months, SD 1.72
4 months	Lip surgery: Millard technique Mean age 3.9 months, SD 0.50	Lip surgery: Millard technique Mean age 4.4 months, SD 0.55	Lip surgery: Millard technique Mean age 4.4 months, SD 0.27
12 months	Soft palate closure: von Langenbeck technique Mean age 11.8 months, SD 0.99	Soft palate closure: von Langenbeck technique Mean age 12.6 months, SD 1.18	Soft palate closure: von Langenbeck technique Mean age 12.6 months, SD 1.14
9 years	Hard palate closure with alveolar bone grafting: modified Hall technique (chin and crista iliaca) Mean age 8.8 years, SD 0.67	Hard palate closure with alveolar bone grafting: Boyne and Sands (chin) Mean age 10.2 years, SD 1.12	Hard palate closure with alveolar bone grafting: von Langenbeck technique (crista iliaca) Mean age 9.8 years, SD 0.83

*SD* standard deviation

**Fig. 1:**
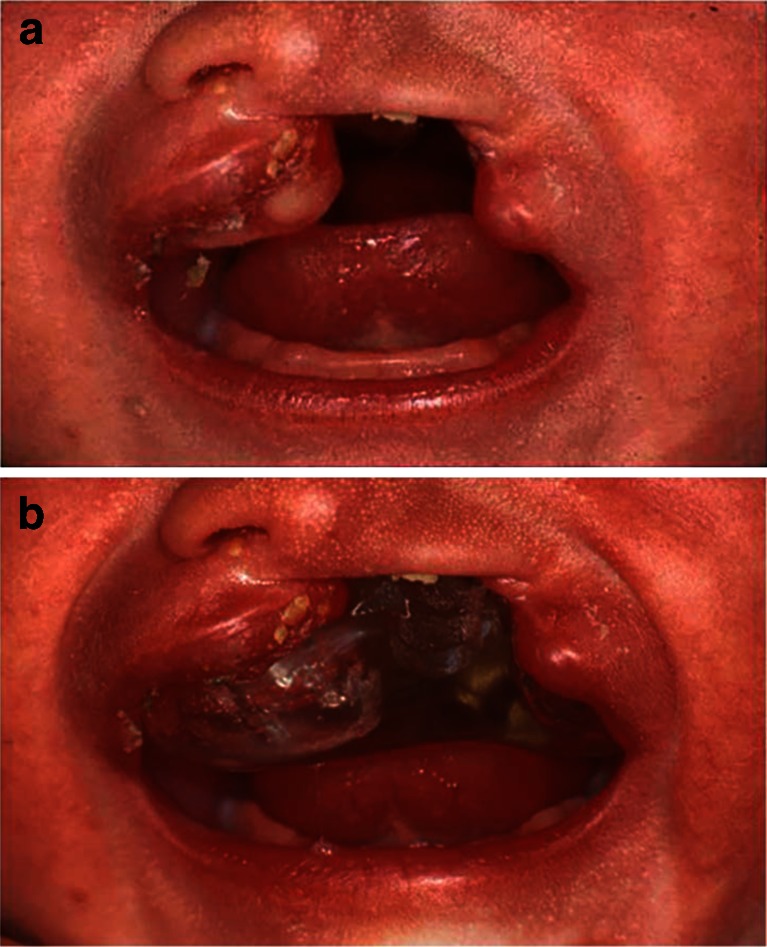
Patient with UCLP with (**a**) and without (**b**) plate

Half of the patients (*n* = 27) were treated with infant orthopedics (IO+) by means of passive plates, starting within 2 weeks after birth, until surgical soft palate closure (Fig. 1a, b). The plate was fabricated on a plaster cast and consisted of compound soft and hard acrylic. The plate was placed in situ within a few days after the impression and worn 24 h a day, except for cleaning. IO+ children returned to the clinic every 3 weeks to have their plates adjusted by grinding at the cleft margins to ensure proper approximation of the maxillary segments. Maxillary growth and emergence of deciduous teeth indicated the necessity for a new plate. Any broken or missing plate was repaired or replaced. After surgical lip closure, the plate was relieved in the frontal area and re-inserted the same day. Check-up visits were now planned every 4 to 6 weeks. The plate was worn until surgical closure of the soft palate.

Children not treated with infant orthopedics (IO−) group did not wear plates. These infants visited the clinic for an extra checkup at the age of 6 weeks as well as before and after lip repair and soft palate closure.

Most of the children (*n* = 35) received orthodontic treatment before closure of the hard palate which was indicated on an individual base. Treatment consisted of expansion and/or alignment of the upper dental arch with fixed or removable appliances such as a quad-helix, RME, TPA, removable (expansion)plate, or fixed appliances.

### Surgeons and orthodontists

Each center had one or two experienced surgeons to perform CLP surgery. The participating surgeons had 7 to 30 years experience in CLP surgery at the onset of this trial. The three participating orthodontists had 6 to 28 years experience with IO. The mean annual volume of children with clefts (including associated malformations) of each center during the intake period (1993–1996) was as follows: Amsterdam 25 infants, Nijmegen 56 infants, and Rotterdam 46 infants. It was aimed to involve only three surgeons, one in each centre. Due to retirement and other circumstantial reasons, the total number of surgeons turned out to be seven (lip surgery), of whom five surgeons were involved in soft palate surgery. The main team surgeons performed the majority (88 %) of the operations. The maxillofacial surgeons performed the bone grafting.

### Data acquisition

Impressions were taken at about 9 years of age (T1) and at 12 years of age (T2), and plaster casts were made. Impression material was chosen based on the presence of an oronasal communication. Alginate impression material (Orthotrace®, Cavex Holland, Haarlem, The Netherlands) was used in case of complete closure and an elastomeric precision material (Lastic®, Kettenbach, Eschenburg, Germany) in case of oronasal communication. The casts were blinded by recoding them.

**Fig. 2:**
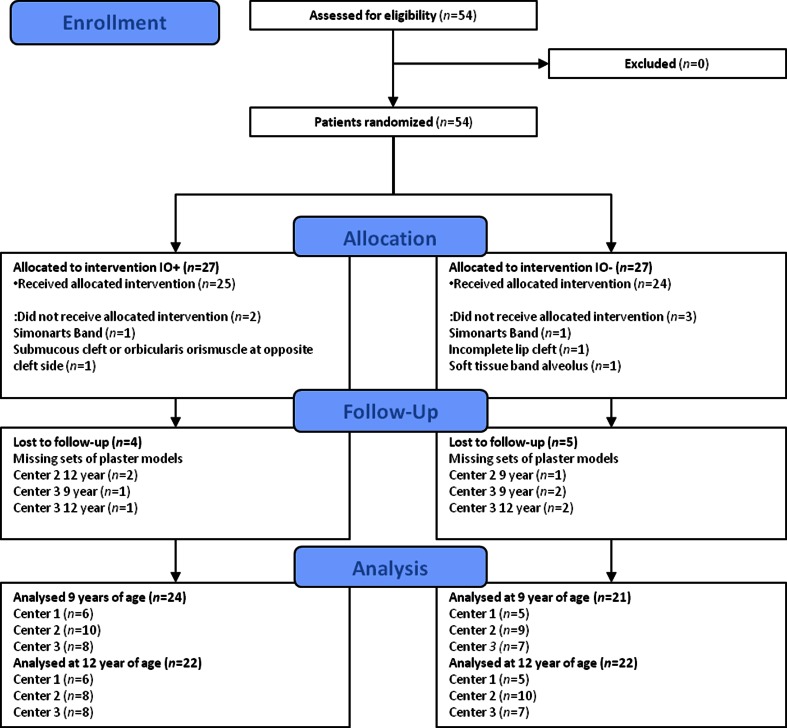
CONSORT flowchart of patients through the trial and reasons for exclusion of evaluation

Nine sets of casts were missing or were never made (Fig. 2).

### Methods

**Fig. 3:**
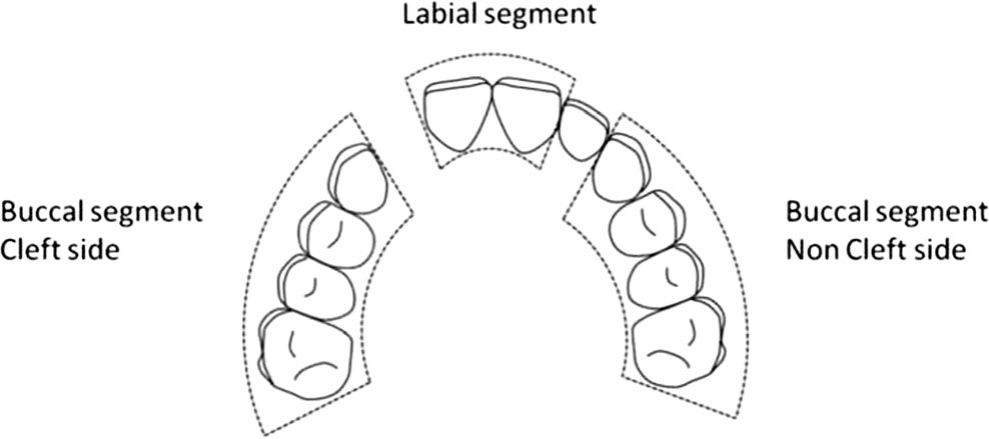
Segmental division of the maxillary arch

**Fig. 4:**
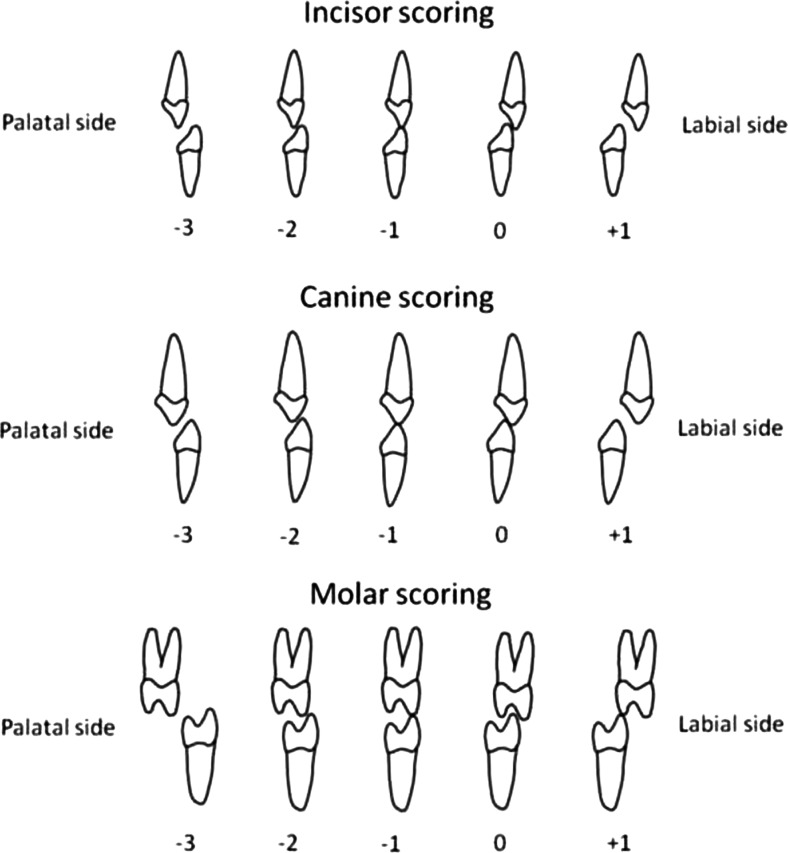
Scoring of the transverse dental relationship

The primary outcome variable “transversal dental arch relationship” was analyzed in the late mixed and/or early permanent dentition, using the Huddart/Bodenham system. [21] The dentition is divided into three segments, a labial, a greater buccal (non-cleft), and a lesser buccal (cleft side) segment. In the labial segment, the lateral incisors are not assessed, as they are frequently missing or unreliable in their position (Fig. 3). First molars are not included before the age of 6 years. To reflect the maxillary arch constriction for the ages of 9 and 12 years, the modified Huddart/Bodenham system is used requiring scoring of the first permanent molar to first permanent molar. Premolars and first molars were scored in the same way as primary molars. Each maxillary tooth or molar was given a score from −3 to +1, depending on its relationship with the corresponding tooth in the mandible (Fig. 4). The modified Huddart/Bodenham score for a given model is described as the sum of the scored teeth, “total arch constriction score,” with a range of the score −30 to +10.

Rules were agreed for situations where there might be ambiguity:

1. If a central incisor was missing, the other central incisor score was used.
2. Where primary canines were missing, the score was determined by the midpoint of the alveolar ridge.
3. If a (primary) molar was absent, then a score was allocated equivalent to the adjacent molar, if it exists. Where both molars were absent, the score was determined by the midpoint on the alveolar ridge.
4. At 5 years of age, the first permanent molars were not erupted and not scored; therefore, the maximum range of scores was −24 to +8.
5. If a central incisor of canine had an extreme rotation, the score was determined by the transverse relationship of the midpoint of the incisal edge.
6. In case of buccolingual angulation of a tooth, the score was determined by the buccolingual position of the root in the alveolar arch.

### Examiners

Three examiners scored the recoded and blinded models independently using the modified Huddart/Bodenham system. The examiners were blinded for treatment. Examiners B and C were experienced orthodontists at the cleft palate team of Amsterdam, and examiner A an orthodontic resident. All examiners repeated the scoring under similar conditions 1 month later, to allow calculation of inter- and intra-examiner reliability and minimize the possible influence of memory on the results.

### Statistics

Intra- and inter-examiner agreements were calculated using the intraclass correlation coefficient (ICC) based on the lateral segments, frontal segment, and total arch constriction scores. The ICC is used to assess the consistency of the measurements of moments 1 and 2, which are made by three observers measuring the same quantity.

The measurement error of the frontal segments, lateral segments, and total arch constriction scores was calculated using Dahlberg’s formula [22].

Independent samples *t*-test were done to evaluate the effect of IO, the cleft sidedness, the influence of orthodontic treatment during the study period, and age of hard palate closure with alveolar bone grafting. An ANOVA and a Bonferroni post hoc test were used to evaluate the center-effect at 9 and 12 years of age. The average scores used to perform the *t*-test, ANOVA, and Bonferroni post hoc test consisted of all measurements done per case. A Pearson chi-square test was used to evaluate a possible bias between orthodontic treatment and the use of IO.

Other possible confounding factors were not evaluated because of randomization at trial entrance and the corresponding treatment protocols.

All statistics were done in SPSS Statistics for Windows, Version 17.0. Chicago: SPSS Inc.

## Results

### Subjects

At intake, 54 patients participated in the study. An overview of the sample characteristics is given in Table 2. After randomization, five patients were excluded. The flow diagram in Fig. 2 shows the reasons for non-evaluation. Two IO+ children hardly used the plate; in one case, plates were worn for 78 weeks. These children remained in the IO+ group according the intention to treat principle. The mean duration of IO was 50 weeks (standard deviation (SD) 16 weeks). Mean age and SDs of lip surgery, closure of the soft palate, and closure of the hard palate with alveolar bone grafting are given in Table 1. Nine sets of casts were missing or were never made. The number of patients evaluated in the IO+ and IO− groups at 9 and 12 years of is are presented in Fig. 2.

**Table 2:** Sample characteristics

Variable	IO+ (*n* = 27)			IO− (*n* = 27)		
Gender: male/female (*n*)	20/7			21/6		
Side of cleft: left/right (*n*)	17/10			18/9		
Patients per center: 1/2/3/ (*n*)	7/11/9			7/10/10		
Age: 9-year casts (years.months)	Mean 9.0			Mean 9.0		
	Range 8.9–9.10			Range 8.1–9.5		
Age: 12-year casts (years.months)	Mean 11.5			Mean 11.1		
	Range 10.7–12.8			Range 10.6–13.2		
	IO+			IO−		
	P10	P50	P90	P10	P50	P90
Age at trial entrance (days)	0	3	7	1	6	13
Birth weight (g)	2660	3350	4020	2920	3600	4280
Cleft width at birth (mm)	9.5	12.5	14.4	8.6	12.4	16.4

Some variables are presented in percentiles because of skewness (P10 = 10th percentile, P50 = 50th percentile, P90 = 90th percentile)

*IO+* patients treated with infant orthopedics, *IO−* patients not treated with infant orthopedics

### Measurement error

The intra-rater agreement scores by means of ICCs were calculated for the frontal segments, both buccal segments individually as well as for the total score. These ICC scores at 9 and 12 years of age were all above 0.8, which represents excellent intra-rater agreement between measuring moments 1 and 2 (Table 3). For all pairs of examiners, the inter-rater agreement scores were well above 0.8 for 9 and 12 years at both measuring moments. This indicates excellent inter-rater agreement (Table 4).

**Table 3:** ICC scores for intra-rater agreement for the total modified Huddart/Bodenham score and the modified Huddart/Bodenham score of the buccal left, buccal right, and front segment

*n* = 45	9 years				12 years			
Rater	Left	Front	Right	Total	Left	Front	Right	Total
A	0.966	0.922	0.967	0.959	0.974	0.965	0.972	0.983
B	0.885	0.922	0.910	0.945	0.909	0.953	0.971	0.960
C	0.931	0.890	0.875	0.938	0.821	0.953	0.969	0.920

**Table 4:** ICC scores for inter-rater agreement at scoring moments 1 and 2 for the total modified Huddart/Bodenham score and the modified Huddart/Bodenham score of the buccal left, buccal right, and front segment

*n* = 45	Raters	9 years				12 years			
Scoring moment		Left	Front	Right	Total	Left	Front	Right	Total
1	A–B	0.962	0.928	0.958	0.947	0.931	0.951	0.947	0.960
	A–C	0.927	0.923	0.891	0.940	0.922	0.965	0.956	0.958
	B–C	0.906	0.911	0.908	0.950	0.896	0.949	0.949	0.958
2	A–B	0.952	0.936	0.964	0.956	0.926	0.961	0.972	0.971
	A–C	0.913	0.922	0.946	0.950	0.849	0.946	0.955	0.915
	B–C	0.937	0.881	0.956	0.960	0.880	0.935	0.947	0.918

The measurement error of the intra-rater total arch constriction scores ranged from 0.99 to 1.82 points (Table 5). The measurement error of the inter-rater scores at moments 1 and 2 ranged from 1.27 to 2.06 points. This indicates excellent inter-rater agreement (Table 6).

**Table 5:** Dahlberg measurement error for intra-rater agreement for the total modified Huddart/Bodenham score and the modified Huddart/Bodenham score of the buccal left, right, and front segment (in points)

*n* = 45	9 years				12 years			
Rater	Left	Front	Right	Total	Left	Front	Right	Total
A	0.52	0.67	0.71	1.29	0.69	0.57	0.54	0.99
B	0.82	0.71	0.65	1.59	0.80	0.61	0.93	1.61
C	1.22	0.82	1.00	1.54	0.70	0.56	1.15	1.82

**Table 6:** Dahlberg measurement error for inter-rater agreement at scoring moment 1 and 2 intra-rater agreement for the total modified Huddart/Bodenham score and the modified Huddart/Bodenham score of the buccal left, right, and front segment (in points)

*n* = 45	Raters	9 years				12 years			
Scoring moment									
		Left	Front	Right	Total	Left	Front	Right	Total
1	A–B	0.85	0.76	0.71	1.49	0.98	0.67	0.85	1.56
	A–C	1.22	0.81	1.07	1.55	0.85	0.56	0.88	1.52
	B–C	1.20	0.74	1.16	1.27	0.97	0.62	0.97	1.52
2	A–B	0.88	0.75	0.77	1.54	0.78	0.62	0.97	1.58
	A–C	0.71	0.79	0.99	1.32	0.85	0.65	1.10	1.89
	B–C	0.92	0.84	0.90	1.50	0.97	0.68	1.07	2.06

### Transverse dental arch relationship

Table 7 shows the total arch constriction of the IO− and IO+ groups at 9 and 12 years of age. Results of the independent samples *t*-test indicated that there were no significant differences in total arch constriction between the IO+ and IO− group at both ages. The maximum difference between the mean scores was 0.51 point, at 12 years of age. No harmful side effects of treatment with IO have been found.

**Table 7:** Total arch constriction score divided by IO+/IO−

		*N*	Mean	Mean diff	SD	95 % CI of the difference		Range	*P*
						Lower	Upper		
9 years	IO−	21	−3.02		3.91	−4.80	−1.23	−11 to +2	
	IO+	24	−3.38		4.35	−5.21	−1.54	−13 to +3	
	Total	45		0.36	1.24	−2.13	−2.84	−13 to +3	0.774
12 years	IO−	22	−3.60		4.75	−5.71	−1.49	−17 to +3	
	IO+	22	−4.11		5.29	−6.46	−1.77	−18 to +4	
	Total	44		0.51	1.51	−2.54	3.57	−18 to +4	0.736

*N* number of patients, *Mean* mean total arch constriction score (points), *Mean diff* mean differences, *SD* standard deviation, *95 % CI* 95 % confidence interval of the difference

Differences between the buccal cleft side and the buccal non-cleft side scores were significant at both 9 and 12 years of age (Table 8). The mean difference at 9 years of age was 2.20 points, and at 12 years of age, the mean difference was 2.43 points. The cleft side showed a higher frequency and severity of crossbites compared to the non-cleft side at both ages (Table 8). Table 9 shows the total arch constriction score divided by cleft and non-cleft sides and infant orthopedics. No significant differences were found between the IO− and IO+ group at 9 and 12 years of age.

**Table 8:** Comparison of the buccal cleft side and the buccal non-cleft side

		*N*	Mean	Mean diff	SD	95 % CI of the difference		Range	*P*
						Lower	Upper		
9 years	Cleft side	45	−2.54		2.74			−10 to +3	
	Non-cleft side	45	−0.34		1.65			−6 to +4	
	Total	45		−2.20	3.29	−3.19	1.21	−10 to +4	0.000*
12 years	Cleft side	44	−2.98		2.70			−10 to +1	
	Non-cleft side	44	−0.55		1.75			−6 to +2	
	Total	44		−2.43	2.90	−3.31	1.55	−10 to +2	0.000*

*N* number of patients, *Mean* mean constriction score for the cleft or non-cleft side (points), *Mean diff* mean differences, *SD* standard deviation, *95 % CI* 95 % confidence interval of the difference

**Table 9:** Constriction score of the buccal cleft and non-cleft side divided by the use of IO+/IO−

		*N*	Mean	Mean diff	SD	95 % CI of the difference		Range	*P*
						Lower	Upper		
9 years									
Cleft side	IO−	21	−2.71		2.61			−8 to +2	
	IO+	24	−2.40		2.89			−10 to +3	
	Total	45		−0.31	2.74	−1.98	1.36	−10 to +3	0.710
Non-cleft side	IO−	21	−0.34		1.93			−6 to +4	
	IO+	24	−0.33		1.41			−3 to +2	
	Total	45		0.01	1.65	−1.02	1.00	−6 to +4	0.986
12 years									
Cleft side	IO−	22	−2.97		2.53			−9 to +1	
	IO+	22	−3.00		2.91			−10 to +1	
	Total	44		0.03	2.70	−1.63	1.70	−10 to +1	0.971
Non-cleft side	IO−	22	−0.55		1.64			−4 to +2	
	IO+	22	−0.56		1.88			−6 to +2	
	Total	44		0.02	1.75	−1.06	1.09	−6 to +2	0.977

*N* number of patients, *Mean* mean constriction score for the cleft or non-cleft side (points), *Mean diff* mean differences, *SD* standard deviation, *95 % CI* 95 % confidence interval of the difference

When evaluating effects on the total arch constriction, significant differences were found between centers at 12 years of age (Table 10). This significance was not demonstrated at the age of 9 years.

**Table 10:** Total arch constriction score per center

Age	Center^a^	*N*	Mean	SD	95 % CI		Range	*P*
					Lower	Upper		
9 years	Center 1	11	−3.09	4.71	−6.25	0.07	−11 to +2	
	Center 2	19	−4.72	4.37	−6.82	−2.62	−13 to +2	
	Center 3	15	−1.38	2.46	−2.74	−0.01	−6 to +3	
	Total	45	−3.21	4.11	−4.44	−1.97	−13 to +3	0.059
12 years	Center 1	11	−1.71	4.60	−4.80	1.38	−10 to +4	
	Center 2	18	−6.56	5.34	−9.22	−3.91	−18 to +1	
	Center 3	15	−2.18	3.19	−3.95	−0.41	−7 to +3	
	Total	44	−3.86	4.97	−5.37	−2.34	−18 to +4	0.008*

*N* number of patients, *Mean* mean total arch constriction score (points), *SD* standard deviation, *95 % CI* 95 % confidence interval of the difference

^a^The difference between age 9 and 12 years was tested for each center by means of an ANOVA. No significant statistical differences were found

The differences between centers in mean scores and standard deviations are greater than the differences found between the IO−/IO+ groups, implying a variation amongst the centers. The range of the scores also showed a difference at both 9 and 12 years of age. This is mainly due to the different minimum scores between the centers. The constriction score of all centers, center 1 showed no clear trend between 9 and 12 years of age (Table 10).

The Bonferroni post hoc test was done to evaluate the centers in couples. Table 11 shows there are no significant differences between the three centers at 9 years of age. At 12 years of age, the mean constriction score of center 2 differs significantly from centers 1 and 3, and the scores of centers 1 and 3 are in concordance.

**Table 11:** Evaluation of the total arch constriction in pairs

	Center	Center	Mean	95 % CI		*P*
				Lower	Upper	
9 years	1 1 2	2	1.63	−2.09	5.34	0.842
		3 3	−1.71 −3.34	−5.61 −6.73	2.18 .05	0.836 0.054
12 years	1 1 2	2	4.85	0.53	9.17	0.023*
		3 3	0.47 −4.39	−4.02 −8.33	4.95 −0.44	1.000 0.025*

*Mean* mean total arch constriction score (points), *95 % CI* 95 % confidence interval of the difference

Presence or absence of orthodontic treatment in the deciduous dentition or mixed dentition before or after bone grafting had no influence on the arch constriction score. The Pearson chi square test gave no indication for possible bias caused by indicating the orthodontic treatment (*P* = 0.676). Table 12 shows the total arch constriction scores sorted by orthodontic treatment at the age of 9. Orthodontic treatment does not influence the outcome of IO at the age of 9 and 12 years. As a possible confounder, sidedness of the cleft was evaluated. Sidedness of the cleft has no influence on the outcome of IO at the age of 9 years (*P* = 0.412) and 12 years (*P* = 0.429). Furthermore, age of hard palate closure with alveolar bone grafting was analyzed. Age of closure differs significantly between centers 1, 2, and 3 (Table 13). The Bonferroni post hoc test showed center 1 had a significant lower age at closure compared to centers 2 and 3 (Table 14).

**Table 12:** Total arch constriction score divided by the use of orthodontic treatment in the deciduous, early, and late mixed dentition

		*N*	Mean	Mean diff	SD	95 % CI of the difference		Range	*P*
						Lower	Upper		
9 years	Treatment −	17	−3.37		4.02			−11 to 3	
	Treatment +	26	−2.97		4.01			−13 to 2	
	Total	43		−0.40	1.25	−2.93	2.13	−13 to 3	0.752
12 years	Treatment −	17	−4.12		4.30			−11 to 3	
	Treatment +	26	−3.19		4.84			−18 to 4	
	Total	43		−0.93	1.44	−3.85	1.99	−18 to 4	0.523

*N* number of patients, *Mean* mean total arch constriction score (points), *Mean diff* mean differences, *SD* standard deviation, *95 % CI* 95 % confidence interval of the difference

**Table 13:** Age of hard palate closure with alveolar bone grafting divided by center

	*N*	Mean age of closure	SD	95 % CI		Range	*P*
				Lower	Upper		
Center 1	11	8.85	0.67	8.39	9.30	8.1 to 10.4	
Center 2	19	10.40	1.12	9.86	10.94	9.4 to 14.6	
Center 3	18	9.84	0.83	9.43	10.25	8.2 to 11.3	
Total	45	9.83	1.09	9.52	10.15	8.1 to 14.6	0.000

*N* number of patients, *Mean* mean age of hard palate closure with alveolar bone grafting (years), *SD* standard deviation, *95 % CI* 95 % confidence interval of the difference

**Table 14:** Evaluation of age of hard palate closure with alveolar bone grafting in pairs

Center	Center	Mean diff	95 % CI		*P*
			Lower	Upper	
1	2	−1.55	−2.43	0.68	0.000
1	3	−0.99	−1.88	−0.11	0.023
2	3	0.56	−0.20	1.32	0.218

*Mean diff* mean difference in age of hard palate closure with alveolar bone grafting (years), *95 % CI* 95 % confidence interval of the difference

## Discussion

In spite of all research in the past decades regarding the claimed advantages and disadvantages, IO still remains controversial. Papadopoulus et al. [14] and Uzel et al. [15] concluded in their reviews that existing evidence could not support any short- or long-term effectiveness of IO in UCLP patients. Earlier treatment outcome of the DUTCHCLEFT trial is comparable with results of the best Eurocleft centers, which did not use IO,[23] and showed that the effects of IO with passive plates as performed in this study did not last beyond the surgical soft palate closure, nor prevented collapse of the alveolar segments at the age of 18 months [24]. IO at the age of 4 and 6 years did not influence the occlusion and maxillary arch dimensions of the deciduous dentition [13, 17–19]. Therefore, the authors of these papers state there is no orthodontic need to perform this type of IO on children with UCLP.

In the present study, the focus was on the transversal dental arch relationship, and the modified Huddart/Bodenham system was used to evaluate the effect of IO on the transverse dental arch relationship at the age of 9 and 12 years. It measures the upper dental arch constriction of children with UCLP by means of the frequency and severity of crossbites in the labial and lateral segments. The scoring system used was designed for the primary dentition and was modified to be used in the transitional and permanent dentition. Therefore, it can be used for any age from 3 years upwards. Beside this flexibility, other advantages are the simplicity (easy to learn and apply), the objectivity, and the sensitivity. The Huddart/Bodenham system appeared to be a valid and reliable indicator of treatment outcome for patients with UCLP [21, 25]. This is emphasized by the fact that the Huddart/Bodenham system measures the transversal dimension by means of three segments, compared to the GOSLON score, which is mainly an anterior-posterior outcome and is not dependent on reference models. Furthermore, the Huddart/Bodenham system measures mainly the outcome of surgery and, in contrast with the GOSLON score, is also a reflection of the iatrogenic damage. Due to the continuous score, the modified Huddart/Bodenham system is more powerful, compared to the ordinal GOSLON score [25, 26].

As could be expected, because no previous differences were found, this study shows comparable results concerning the effect of IO on the transverse dental arch relationship in children with UCLP as reported by Prahl et al. and Bongaarts et al. for the same sample at a younger age [13, 17, 18, 24]. Scores of the total arch constriction of patients treated with and without IO did not differ significantly at both 9 and 12 years of age. These findings are logical and in concordance with earlier findings since one cannot expect a treatment not showing a significant treatment effect at the age of 18 months having an effect at 9 or 12 years of age.

Patients, regardless of IO treatment, have more crossbites on their buccal cleft side segment compared to their buccal non-cleft side at 9 and 12 years of age. Crossbites are the clinical expression of the transverse dental arch relationship measured by the Huddart/Bodenham score. Unfortunately, little attention has been given to this topic in recent literature. Orthodontists could take this finding into account in their treatment planning in children with UCLP.

At 9 years of age, differences in the transverse dental arch relationship between the three centers are larger than the differences found between the IO+ and IO− group. These differences are not significant. However, at 12 years of age, these differences are significant. Centers 1 and 3 have significantly different results compared to center 2. These significant differences are larger than the calculated measurement error. The total arch constriction score of all centers show no clear trend between 9 and 12 years of age (Table 10). Besides these differences in mean scores, the range of the scores at both ages is larger in center 2 and mainly due to lower minimum scores in centers 1 and 3.

Until the age of 1.5 years of age, consensus was reached on timing and type of the surgical interventions. The surgeons standardized their surgical techniques and the orthodontists their follow-up. Randomized clinical trials are time-consuming and take a lot of effort of the patient and the team members. It is even questionable if it is ethical to treat children with UCLP according to a strict protocol throughout their entire childhood. Individualizing a treatment protocol that meets the individual needs of the patient could ensure an optimal treatment. As a consequence, cofactors influencing the outcome are unavoidable and illustrate the limitations of this study.

Differences between centers could not be explained by the randomization criteria birth weight and cleft width of the cleft at trial entrance and corresponding treatment protocols until the age of 1.5 years these. Treatment after 1.5 years of age could possibly have an effect. The hard palate of all patients was closed simultaneously with bone grafting according to the protocol of all teams. Due to the late closure of the hard palate, there was time for the maxilla to develop three-dimensionally compared to other studies without late closure of the hard palate. Therefore, results of the present study always have to be compared with care. Age of hard palate closure with alveolar bone grafting differed significantly between the participating centers, although not more than 1.5 years (Table 1). This finding had no clinical impact since the time span from the closure of the hard palate until the evaluation at 12 years of age is probably too short to have any detrimental effect. Orthodontic treatment before and just after closing of the hard palate did not influence the transverse dental arch dimensions at 9 and 12 years of age (Table 11). Type and frequency of orthodontic treatment in the deciduous, early, and late mixed dentition differed between the participating orthodontic centers, and it is still unclear what the influence is of the remaining cofactors not investigated. Therefore, more long-term research has to be done.

## Conclusions

Transverse dental arch relationships at 9 and 12 years of age do not differ between children with UCLP treated with IO or not. Therefore, there is no orthodontic need to perform IO as applied in this study in children with UCLP.

The mean differences in the transverse dental arch relationship are significant at 12 years of age. This could not be explained by any factor taken into account in this study. More prospective long-term research is needed on the side effects of other cofactors such as surgery in children with UCLP.
